# Clinical evaluation of SARS-CoV-2 lung HRCT and RT-PCR Techniques: Towards risk factor based diagnosis of infectious diseases

**DOI:** 10.1016/j.csbj.2021.04.058

**Published:** 2021-04-30

**Authors:** Fariba Asadi, Razieh Shahnazari, Nikhil Bhalla, Amir Farokh Payam

**Affiliations:** a Fateme Alzahra Hospital, Isfahan University of Medical Sciences, Isfahan, Iran; b Firozabadi Hospital, Department of Radiology, School of Medicine, Iran University of Medical Sciences, Tehran, Iran; c Nanotechnology and Integrated Bioengineering Centre (NIBEC), School of Engineering, Ulster University, United Kingdom; d Healthcare Technology Hub, Ulster University, BT37 0QB Jordanstown, United Kingdom

**Keywords:** COVID-19, Coronaviruses, RT-PCR, HRCT, Age, Gender

## Abstract

This study uses image analysis techniques for comparative analysis of the lung HRCT features and RT-PCR of 325 suspected patients to COVID-19 pneumonia. Our findings propose more caution in the interpretation of RT-PCR data, promoting, instead, also the quantification of age and sex-based risk factors using HRCT images. Statistical analysis of our methodology reveals a direct relation between intensity, skewness and kurtosis of the radiological features and the gender of patients. Moreover, we investigate the effect of the age of patients on the appearance of COVID-19 pneumonia in the HRCT images. We have also applied our methodology to investigate the effect of time on the severity of COVID-19 pneumonia within the lungs. Subsequently, we find a strong relationship between image analysis and the informed medical diagnosis asserted by the radiologists. Additionally, our results also indicate increase in the severity of lung infection in the first and second week after the onset of the SARS-CoV-2 symptoms. Thereafter, a gradual decrease in the lung damage is observed during the third week. The proposed image analysis methodology can be used as a simple complementary tool for infectious disease diagnostics as demonstrated in this study with an example of SARS-CoV-2 to provide better understanding of the disease for drug and vaccine development.

## Highlights

• Image analysis of HRCT and PCR of 325 patients.
• HRCT and PCR should be used as a complementary tool for the detection of COVID-19.
• Age and gender act as relevant risk factors in SARAS-CoV-2 infection.
• Risk assessment should be a part of COVID-19 treatment.

## Graphical abstract

**Figure:**
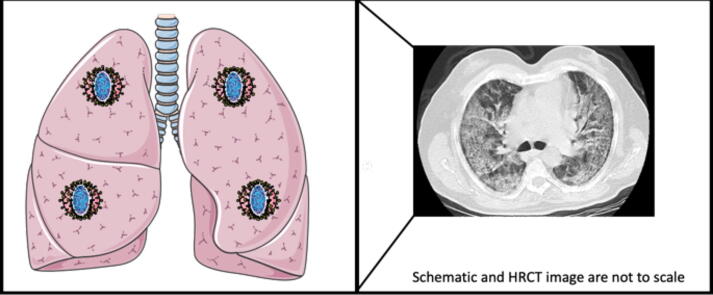

## 1 Introduction

In December 2019, Severe Acute Respiratory Syndrome of Coronavirus 2 (SARS-CoV-2) appeared in Wuhan, China and shortly spread in the whole world [1]. Fever, dry cough, dyspnea, chest tightness and shortness of breath are common symptoms of SARS-CoV-2 which can lead to severe injury in the lungs and spreading of the virus to other organs such as kidneys, heart, thyroid, and adipose tissue [2], [3], [4], [5], [6]. In particular, SARS-­CoV-­2 infect human respiratory epithelial cells through the interaction of viral protein and the Angiotensin Converting Enzyme 2 (ACE2) receptors abundantly present on the cells forming the epithelial layers of the human respiratory tract [4], [6], [7], [8], [9], [10], [11]. Several factors such as age, sex and comorbidities lead to variation in complications associated with COVID-19 pneumonia, ranging from asymptomatic cases to patients with severely damaged lungs. [12], [13], [14]. The repercussions of these symptoms and associated risk factors can lead to health complications in long term and in many cases these symptoms can be fatal in short term, if left unaddressed. As such the development of intervention medicine (e.g. specific drug) is still in pre-mature stage, while vaccination may have cause unwanted changes in the body in long term [15]. Therefore, precise detection and continuous informed monitoring of COVID-19 infection is urgently required to be established, one possible route can be through the customization/improvement in analysis of techniques routinely used in medical industry.

Current detection methods which are widely used for diagnosis of SARS-CoV-2 are Reverse Transcription Polymerase Chain Reaction (RT-PCR), chest X-ray, High Resolution Computed Tomography (HRCT) scans, and the detection of some common biomarkers in the blood [16], [17], [18], [19], [20]. However, recent studies reported that the positive rate of RT-PCR is between 30% and 60% [20], [21], [22], [23]. This suggests that at initial stage of the disease, many infected cases may not be detected due to the low sensitivity of RT-PCR. In parallel, recent clinical investigations have revealed the advantages of Chest-HRCT (hereafter referred as HRCT) to demonstrate typical radiological features in the patients suspected to COVID-19 pneumonia, consisting both positive and negative RT-PCR results [20], [22], [23], [24], [25], [26]. However, due to the infancy of the research in the HRCT and RT-PCR analysis of COVID-19 pneumonia, in addition to the limitation of HRCT such as low specificity, need of high level skills (combination of clinical and image development expertise) in HRCT image analysis and low sensivtity of HRCT for early stage rapid disease detection [27], [28], [29], new and simple image analysis are required to enhance the capaciaty of HRCT and RT-PCR. For instance, understanding the association between risk factors and the findings of the HRCT in combination with RT-PCR may lead to more specific detection than the current state of art in the use of HRCT and RT-PCR for disease detection.

In this context, we report clinical results of HRCT and RT-PCR of 325 patients suspected with COVID-19 pneumonia and evaluate the advantages of HRCT in comparison to the RT-PCR test. Furthermore, based on the quantitative and statistical image analysis on the HRCT data, we found the relation between sex and age as main risk factors in the enhancement of the disease and features identified in the HRCT.

In order to investigate our hypothesis, RT-PCR and HRCT was conducted in the patients and new image analysis was performed in the patient data. Starting from HRCT outcomes, RT-PCR results have been analyzed and associated to the assertions made using image analysis. The obtained results are discussed with respect to age and sex-based risk factors to quantify with the severity of damage in the lungs due to the COVID-19 pneumonia.

## 2 Methods

The institutional review board of Iran University of Medical Science have approved this retrospective study and the requirement to obtain a written informed consent was waived. This study has been performed on the suspected patients to SARS-CoV-2 infection who came to Fateme Alzahra hospital in Isfahan province as the reference hospital for COVID-19 disease in Najaf Abad city in the first phase of COVID-19 pandemic (13 March-27 April 2020).

### 2.1 Chest HRCT protocol

All HRCT were performed using a GE scanner (Optima CT540, UK). A low-dose institutional protocol was applied with the main scanning parameters as follows: tube voltage: 100kVp; tube current: 10–202 mAs; automatic exposure control; slice thickness = 3.75 mm. CT images were acquired at full inspiration with the patient in supine position, and without administration of intravenous contrast medium.

### 2.2 RT-PCR

Real-time RT-PCR assay has been performed in the hospital with samples obtained from endotracheal aspirate using nasopharyngeal/oropharyngeal swabs. The RT-PCR kits that were approved by WHO (Name of manufacturer: Sansure, China and Dangene, China) were used for COVID-19 tests. The cycle threshold (CT) value of 39 is used in this work as per the guidelines given the kits. The kits identify nucleocapsid, RNA dependent RNA polymerase (RdRp) and ORF1 viral genes of COVID-19. If one gene was positive, the test was repeated, and then if same gene was detected again, a positive result was reported otherwise from patient test was repeated again to confirm negative/positive outcome.

### 2.3 Image analysis

Two radiologists (F.A. and R.S., with 6 and 7 years of experience in interpreting chest CT images, respectively) blinded to RT-PCR results reviewed all HRCT images and decided on positive or negative HRCT findings by consensus. The epidemiologic history and clinical symptoms (fever and/or dry cough) were available for both readers. The radiologists classified the HRCT scan (according to Radiological Society of North America (RSNA) classification) as, typical, indeterminate, atypical and negative for COVID-19 pneumonia. A description of main HRCT features and lesion distribution has been performed.

### 2.4 Statistical and quantitative image analysis

Quantitative and statistical image analysis was performed using ImageJ software, a java-based image processing tool developed by National Institute of Health (NIH) (https://imagej.nih.gov/ij/download.html). Essentially individual HRCT images were uploaded in the ImageJ software and then converted to 8-bit format. Following image conversion to 8-bit, lung cross section area was selected manually in each image using draw polygon command. This area was then analysed by measuring the average gray value within the selection. Here the measurand is the sum of gray values of all the pixels in the selected area divided by the number of pixels, represented as mean intensity, reported in arbitrary units (a.u.). To check the empirical formula, refer to the documentation of ImageJ in the aforementioned link. This suggests that the presence of cloudy regions in the HRCT image (such as the one corresponding to the ground glass opacity) will show higher values of mean intensity as compared to the normal regions of the lung. It should be noted that higher values of mean intensity imply more damage to the lungs. In addition, we also calculated the kurtosis and skewness of all the gray values within the lung selection area of the HRCT image. The skewness represents the distortion in the normal distribution of the data and kurtosis is a measure of distributions’ tail relative to the centre of the data distribution. If the curve is shifted to the left or to the right from an ideal normal distribution of the data, it is said to be positive or negative skewed respectively. Similarly, if the tail of the data set under analysis extends further than the ideal normal distribution, then it is said to have negative kurtosis and if the tails of the dataset are within the boundaries of an ideal normal curve, it has a positive kurtosis. Here, the physical meaning of skewness and kurtosis can be extended to highlight the severity of the damage in the lungs. In particular, more negative skewness is associated with degree of opacity in the lungs as it indicates more gray pixels in the selected area of the image. Similarly, negative kurtosis indicates that the spread of the gray pixels in the region under selection is wider than the ideal normal distribution, which can be attributed to the large surface covered by the opaque regions in the lungs.

### 2.5 Case selection

**Fig. 1:**
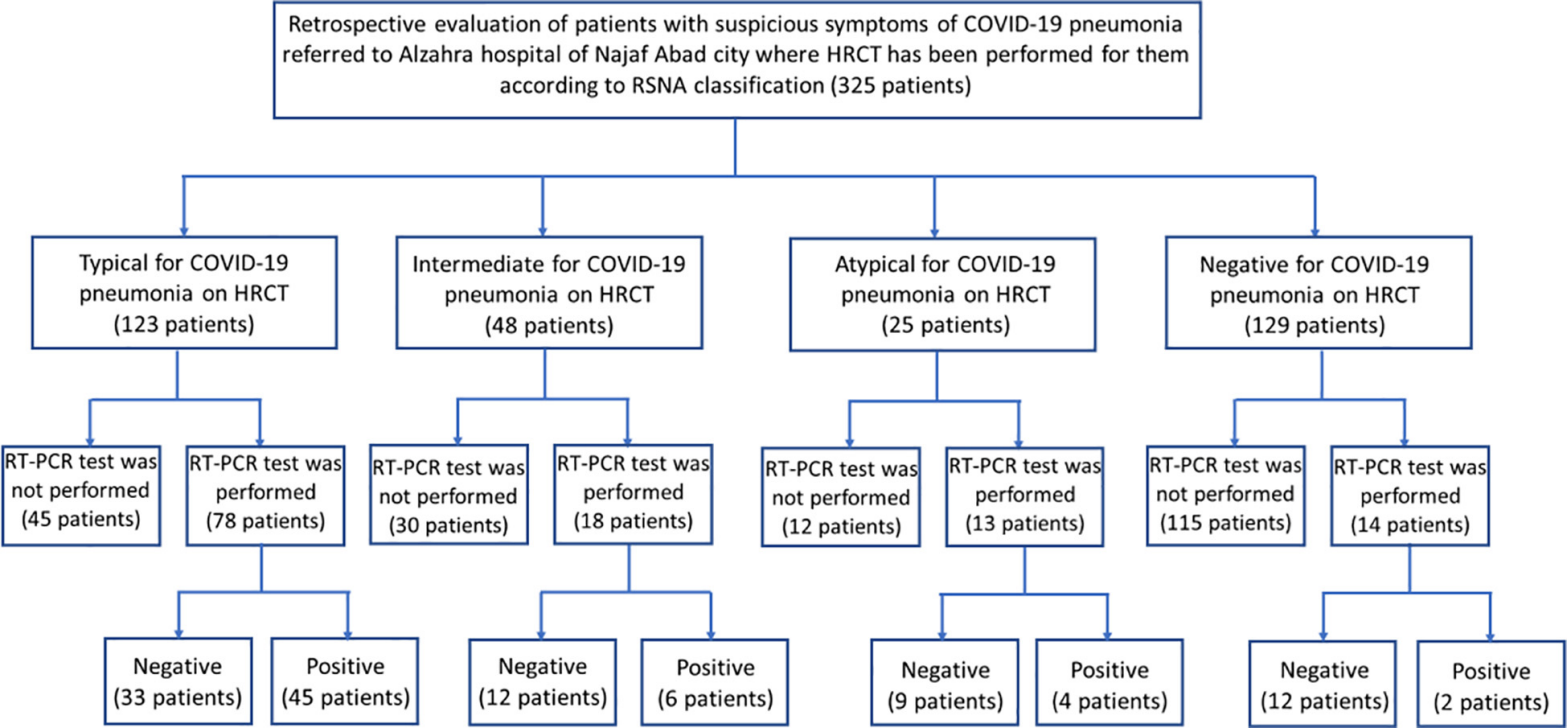
Diagram showing the patient selection for this study.

The diagram of our case selection is given in Fig. 1. In our patients selection we follow Fleischner Society criteria [30]. According to HRCT findings and RSNA classification [31], patients were classified in four groups: patients with typical appearance of COVID-19, intermediate appearance of COVID-19 pneumonia, atypical for COVID-19 pneumonia and negative for COVID-19 pneumonia (other diseases appearance or patients with normal HRCT). Depending on the RT-PCR of the patients within each group, every group is classified into two subgroups: patients for whom RT-PCR was conducted and for whom RT-PCR was not conducted. At the last stage, each subgroup is classified into two sections: patients with positive results of RT-PCR and patients with negative RT-PCR results. However, in this study, retrospective analysis of only RT-PCR positive patients has been performed, and other data of negative RT-PCR test are classified as subsidiary data.

### 2.6 ImageJ study design

**Fig. 2:**
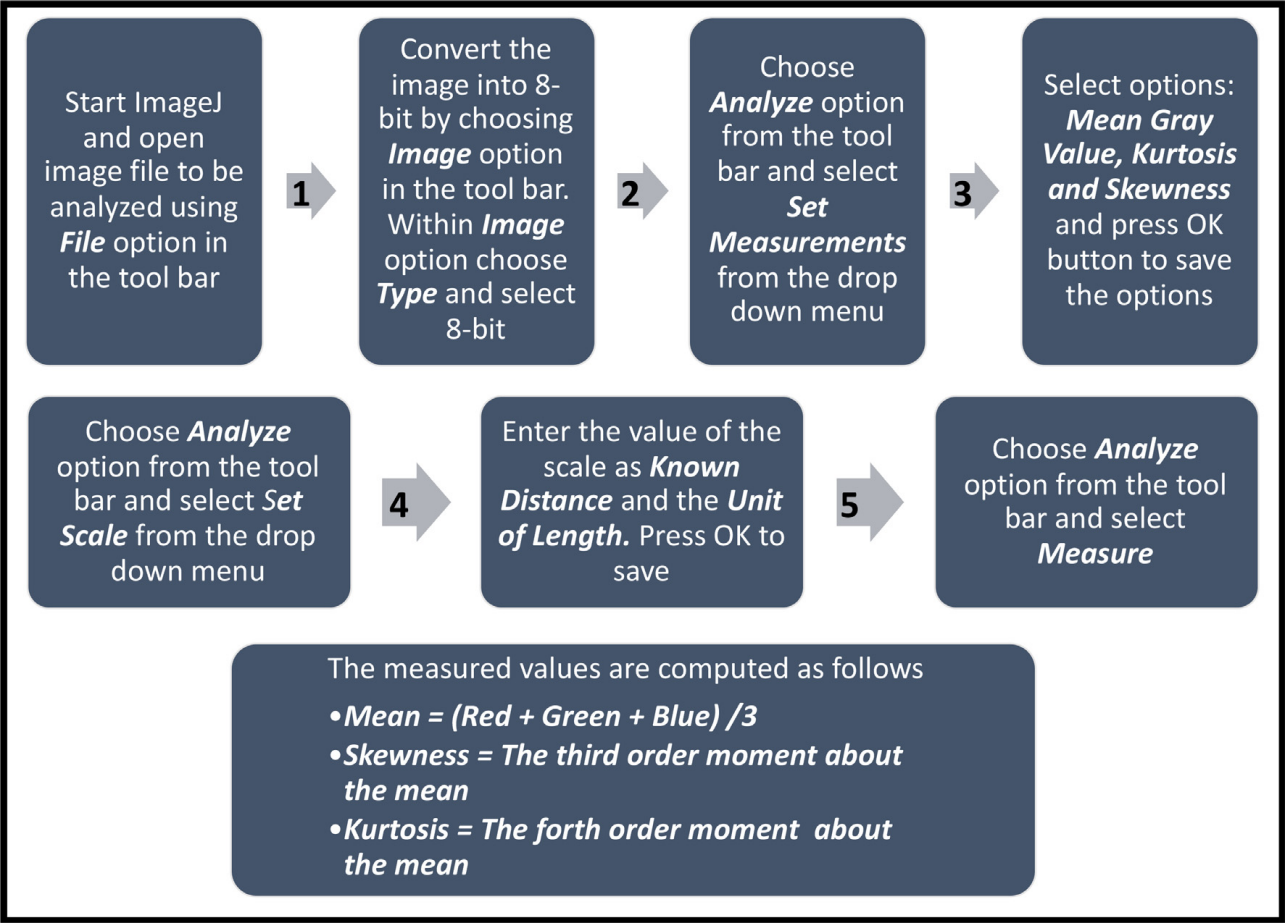
Steps to compute mean, skewness and kurtosis using ImageJ analysis.

General steps involved within the analysis are highlighted in Fig. 2. The flowchart in this scheme shows operation protocol for the analysis of HRCT images and subsequent analysis using parameters of mean, skewness and kurtosis.

It should be noted that the step 5 in the protocol will analyze complete image (including scale bar and text within standard HRCT image) and therefore specific HRCT area for measurement must be manually selected for analysis. This analysis was performed on all HRCT images individually (as each image had different lung sizes) and the data generated was grouped into different categories according to the diagnosis of the radiologists (positive/negative COVID-19, sex and gender). Data from individual categories are reported in the figures within results and discussion section where data variation/errors in each category are reported in form of standard deviations.

## 3 Results and discussions

### 3.1 HRCT and RT-PCR analysis

From 325 patients with low dose HRCT performed on them, 123 cases had typical appearance of COVID-19 pneumonia, out of which 44 cases (35.8%) were women and 79 cases (64.2%) were men. The range of their age was between 24 and 96 years and the mean age was 57.78 years. From these 123 patients 6 of them (4.87%) also had cardiomegaly (3 women, 3 men), 18 patients (14.63%) pleural effusion (2 women ,16 men), 6 patients (4.87%) lung nodule or mass (3 women, 3 men), 3 patients (2.43%) hyperinflation (1 woman ,2 men), 4 patients (3.25%) emphysema or bronchiectasia or fibrosis (1 woman ,3 men) and a man (0.81%) with pulmonary artery dilatation.

Among the 123 infected cases, 18 patients (14.6%) in the range of 34–96 years passed away due to the infection. From the 123 patients which show typical appearance of COVID-19 pneumonia on HRCT, 78 patients were also tested by RT-PCR technique. The RT-PCR test of 45 patients (57.6% of 78 patients) was found to be positive and 33 patients (42.3% of 78 patients) were found to be negative. No RT-PCR test was conducted in the rest of the 45 patients, and they were treated solely based on the lab serology and HRCT data. It should also be noted that in the 45 patients with both positive HRCT and RT-PCR outcomes, 11 patients were women and 34 patients were men with the age range of 28–96 years and mean age of 64.84 years for both sexes. From 325 patients, intermediate HRCT signs were observed in 48 patients, among 18 of them RT- PCR test has been performed and other 30 cases were treated by considering clinical symptoms and serology. From 325 patients, atypical HRCT for COVID-19 pneumonia were observed in 25 patients, among 13 of them RT-PCR test has been performed (4 patients positive) and other cases were treated by considering clinical symptom and serology.

From the pool of all patients (325 patients) 129 patients HRCTs were negative for COVID-19 pneumonia, among them other diseases feature on HRCT including cardiomegaly, hyperinflation, mediastinal mass, emphysematous changes were observed in 68 patients (without imaging evidence of SARS-COV-2 infection) and 61 patients had normal HRCT. However, the RT-PCR test has been performed only for 14 patients out of which 2 patients showed positive results. Table 1 summarize the results.

**Table 1:** Demographic information of patients with typic COVID-19 appearance on HRCT and/or positive results for RT-PCR test.

	Male	Female	Age range (year)	Mean age(year)	Dead patient(number)	Mean age in dead patient(year)
Typical covid-19 appearance on HRCT	79	44	24–96	57.78	18	73.38
Patient with typical covid-19 appearance on HRCT and RT-PCR+	34	11	28–96	64.84	9	76.66
Patient with RT-PCR+ (typical HRCT or not)	42	15	28–96	65.92	11	79.54

Among 325 patients, without considering HRCT results, the RT-PCR test has been performed for 122 patients. In total, 57 patients had positive result in RT-PCR test, where 15 of them were women and 42 patients were men. For both women and men, the age range was 28–96 years, with a mean age value of 65.92 years. Out of 57 patients, 11 patients between 53 and 92 years (mean age: 79.54 years), with positive RT-PCR tests, died.

**Fig. 3:**
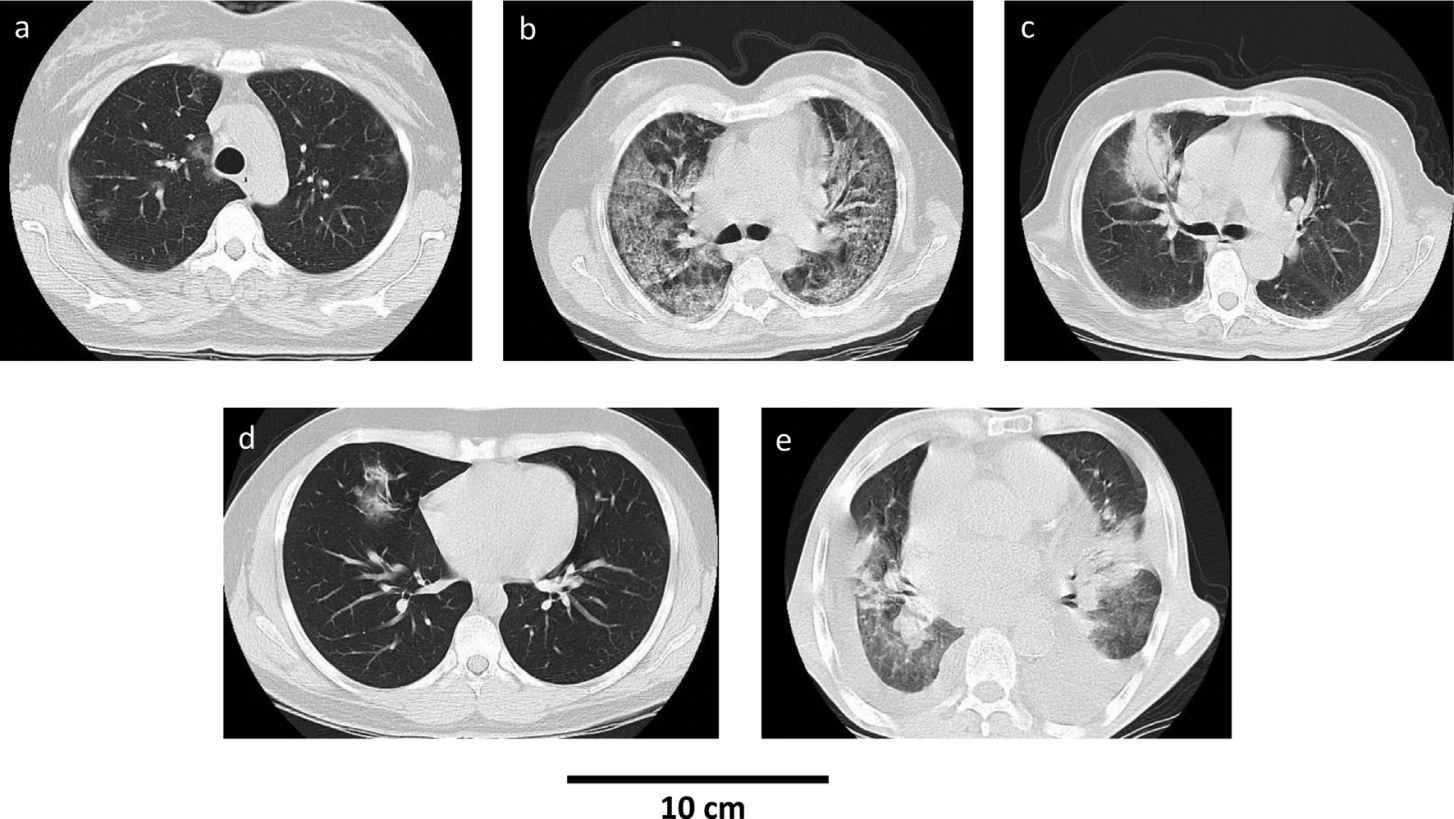
Transverse CT scans a) Low dose lung HRCT shows multiple patchy ground glass opacities in both lungs as sign of COVID-19 pneumonia. b) Low dose lung HRCT shows cardiomegaly and diffuse crazy paving appearance in lungs suggesting COVID-19 pneumonia. c) Low dose lung HRCT shows cardiomegaly and air space consolidation in RML, suggesting lobar pneumonia. d) Low dose lung HCRT shows a consolidation patch in RML with surrounding ground glass opacities (halo sign). e) Low dose lung HRCT showed cardiomegaly, bilateral peribronchiovascular consolidation, pleural effusion, DDX was pulmonary edema and Covid-19 pneumonia. Note for all of cases the results of RT-PCR test are positive.

In this study we focus on the 57 patients, with RT-PCR positive. Among these patients, crazy paving appearance were seen in 18 of them (patchy: 8 patients, continues: 10 patients), Ground Glass Opacities (GGO) is seen in 18 patients, peribronchiovascular involvement has been observed in one patient, consolidation is seen in 13 patients (with air space appearance in 5 of them) and halo sign is observed in 1 patient. In addition, mix appearance is observed in 4 patients, normal HRCT is reported for 1 and cardiomegaly without other involvement has been seen for 1 patient. Fig. 3 shows examples of main CT features observed in the patients with positive RT-PCR test.

We also observed parenchymal involvement in 55 patients using HRCT; in particular the unilateral lung parenchymal involvement was seen in 6 of them while bilateral lung parenchymal involvement is observed in 49 of them. The involvement with anterior dominancy was observed in 9 patients and the involvement with posterior dominancy was seen in 46 patients. The main affected lobe of 12 patients was upper lobe, 5 patients was middle lobe and 38 patients was lower lobe of the lungs. The main infected affected area in 3 patients was in the centre of the lungs, in other 35 patients it was at the peripheral parts of the lung while rest of the 17 patients the infection was continuous in nature. Pleural effusions also existed in 20 patients. Furthermore, cardiomegaly was observed in 5 cases of 57 cases. In 1 patient, pulmonary trunk dilatation was seen.

**Fig. 4:**
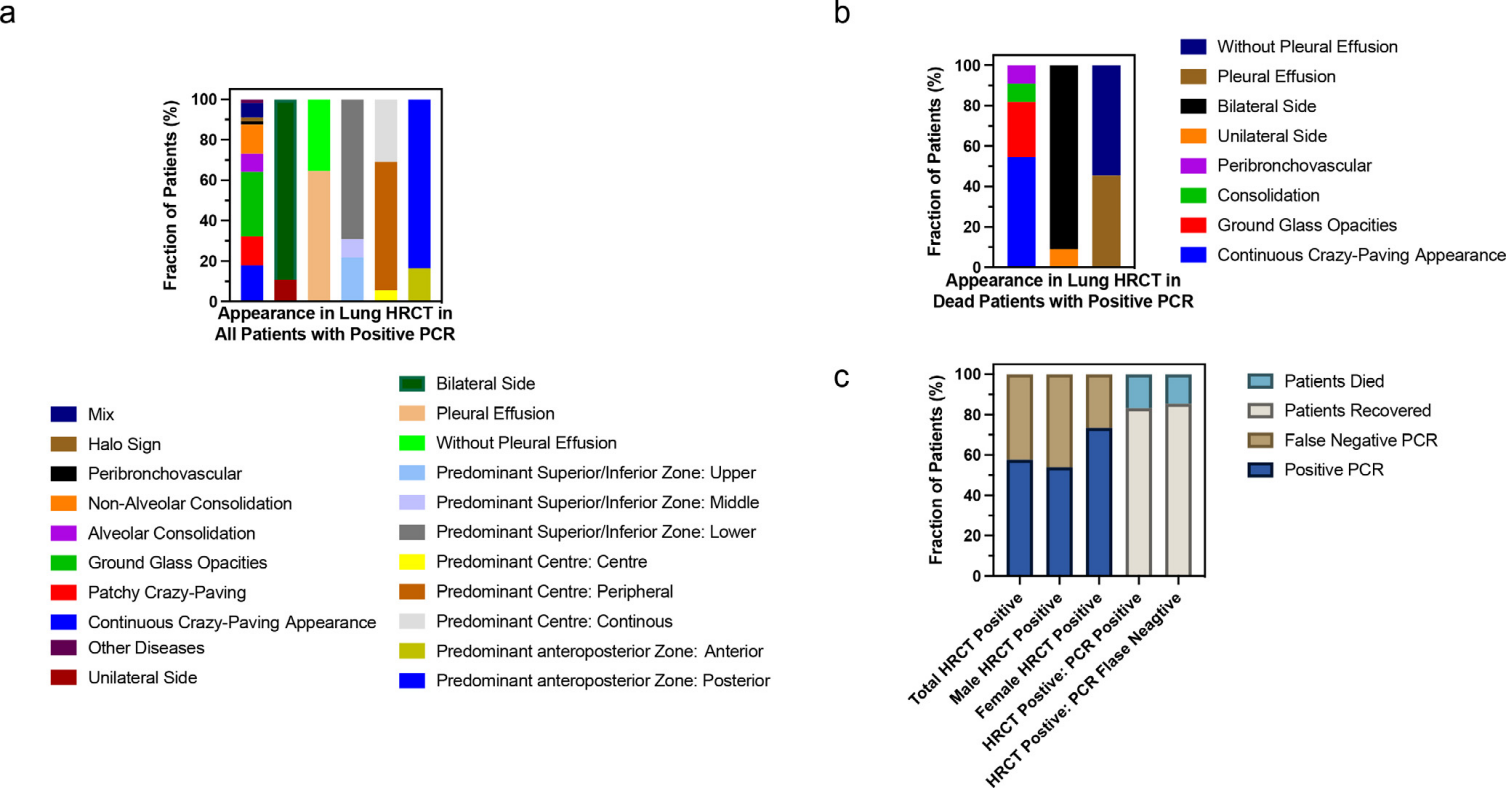
Summary of lung HRCT appearances and patients with positive HRCT. a) Covid-19 appearance on lung HRTC in patients with positive PCR. b) Covid-19 appearance on lung HRCT in dead patients with positive PCR. c) Statistical data of patients with positive HRCT classified based on the gender, recovery/died, positive and negative RT-PCR test results.

Out of 57 patients, 11 patients passed away. In these 11 patients the continuous crazy paving appearance was seen for 6 of them, GGO appearance was observed for 3 of them and the consolidation appearance was seen in one of them. The peribronchovascular was seen in one patient too. Ten patients had bilateral involvement and for 1 dead patient the unilateral involvement was observed and 5 of them had pleural effusion. Fig. 4 and Table 2, Table 3 summarized the appearance of lung HRCT in the patients with positive RT-PCR which recovered (Fig. 4a) or died (Fig. 4b). Fig. 4c summarized the percentage of patients identified as positive HRCT based on their gender and their RT-PCR results (positive or negative). As it can be seen from Fig. 4c, the positive rate of RT-PCR assay is about 55%, which is consistent with that in a previous report [20]–[23]. It means the results of RT-PCR should be interpreted with caution.

**Table 2:** Summary of COVID-19 appearance on lung HRCT in patients with positive PCR test.

COVID-19 appearance on lung HRCT	Number (percentage)
Crazy paving appearance	Continues :10 (17.54%) Patchy :8 (14.03%)
Ground glass opacities	18 (31.57%)
Alveolar consolidation Non-alveolar consolidation	5 (8.77%) 8 (14.03%)
Peribronchiovascular	1 (1.7%)
Halo sign	1 (1.7%)
Mix	4 (7.01%)
Other diseases	1 (1.7%)
Normal	1 (1.7%)
Unilateral side Bilateral side	6 (10.9%) 49 (89.9%)
Predominant superiorferior zone: Upper Middle Lower	12 (21.81%) 5 (9%) 38 (69.09%)
Predominant center Centre Peripheral Continuous	3 (5%) 35 (63.63%) 17 (30.9%)
Predominant anteroposterior zone Anterior Posterior	9 (16.36%) 46 (83.63%)
Pleural effusion	20 (63.36%)

**Table 3:** COVID-19 appearance on lung HRCT in dead patients with positive RT-PCR.

COVID-19 appearance on lung HRCT	Number (percentage)
Continuous crazy paving appearance	6 (54.54%)
Ground glass opacities	3 (27.27%)
Consolidation	1 (9.9%)
Peribrenchiovascular	1 (9.9%)
Unilateral side Bilateral side	1 (9.9%) 10 (90.9%)
Pleural effusion	5 (45.45%)

### 3.2 Risk factor evaluation of lung HRCT features and PCR

**Fig. 5:**
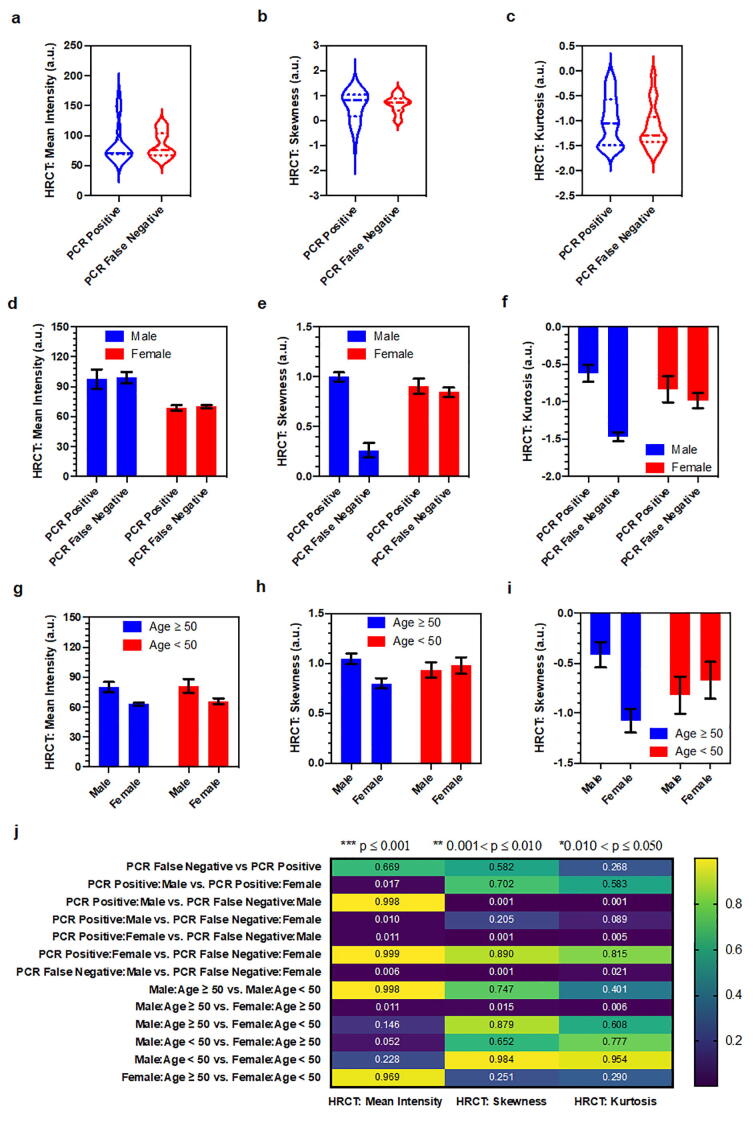
Results for 123 patients which reported all symptoms of COVID-19 infection. All HRCT positive patients for COVID-19 showed mixed results. Images a-c show no statistically significant differences for the whole population at RT-PCR and HRCT scans (without sex & age categorization): a- intensity, b-skewness and c-kurtosis. Images d-f) shows categorization of patients into male and female: d- intensity, e-skewness and f-kurtosis. g-i) segregation of patients into ages<50 and greater than equal to 50: g- intensity, h-skewness and i-kurtosis. j) Shows statistical analysis using *t*-test and 2-way ANOVA (Tukey) performed to highlight statistical relevance of the data. Here, the symbol * indicates the qualitative level of significance (P < 0.05) in the differences of mean values within subfigures a-i.

In order to compare the sensitivity of HRCT with RT-PCR and study the relation between age and gender as important risk factors, quantitative image analysis is performed for the radiological features observed in HRCT. The statistical analysis was computed using non-parametric Mann-Whitney *U* test for data presented in Fig. 5a-c. For all other data multiple comparison, using 2-way ANOVA test, Tukey test with pooled variance is conducted to compare the means of each data test while considering the data as normally distributed within each group set. Two-tailed p value < 0.05 was considered statistically significant, with 95% confidence interval. Fig. 5a shows the statistical results of comparison between cases with positive HRCT and both positive and negative RT-PCR. As seen from results, there is no significant differences between positive and negative RT-PCR in the intensity, skewness and kurtosis calculated from HRCT images. Moreover, it is observed that RNA virus detection cannot completely reply on the RT-PCR as shown by HRCT. RT-PCR negative hits found to be 42.3% for 33 patients out of all 78 patients tested with positive HRCT. It can be explained by the lack of sensitivity, insufficient stability, and relatively long processing of RT-PCR test [20], [32]. Other factors which can affect the accuracy of RT-PCR are specimen source (lower or upper respiratory tract), performance of kits and the sampling time window which is related to the time of disease development with which RT-PCR test has been conducted. In order to study the relation between severity of damages and appearances of COVID-19 pneumonia in the lungs with the gender and age as two main risk factors in SARS-CoV-2 disease [12], [13], [14] , we have performed another image analysis. The results are given in Fig. 5d to j. The HRCT mean (5a), skewness (5b) and kurtosis (5c) show higher intensity values in RT-PCR positive compared to RT-PCR false negative. As it is depicted, generally the mean intensity for males is higher than females independent of RT-PCR results (5d). Furthermore, for both positive and negative RT-PCR results, the mean intensity of males is higher than the females. This is in agreement with recent RNA-sequential and single cells analysis which has been performed on SARS-CoV-2 infected samples to describe higher susceptibility in male than female to COVID-19 pneumonia [4], [33]. According to report of [4], significant correlations between ACE2 expression levels with CD8 + T cell enrichment level (0.20 < r < 0.68) and interferon response signature (0.32 ≤ r ≤ 0.82) in males were detected; on contrary female patients show a negative correlation, r = -0.3 and (-0.26 < r < -0.20), respectively [4]. Moreover, significant differences are observed for false negative PCR in males and HRCT (Fig. 5e and 5f). Based on the definition of skewness and kurtosis it can be deduced that for negative RT-PCR results of male, the cloudy regions in the HRCT images were distributed wider and not concentrated in specific parts of lung. This result can be helpful to understand the reason of false negative RT-PCR results while the appearance of SARS-CoV-2 infection identified in the HRCT images. In addition, comparison of the results associated to the age factor shows that there was no significant difference between appearance of COVID-19 pneumonia in the HRCT images for the patients older and younger than 50 years.

### 3.3 Time-based stages of COVID-19 infection

**Fig. 6:**
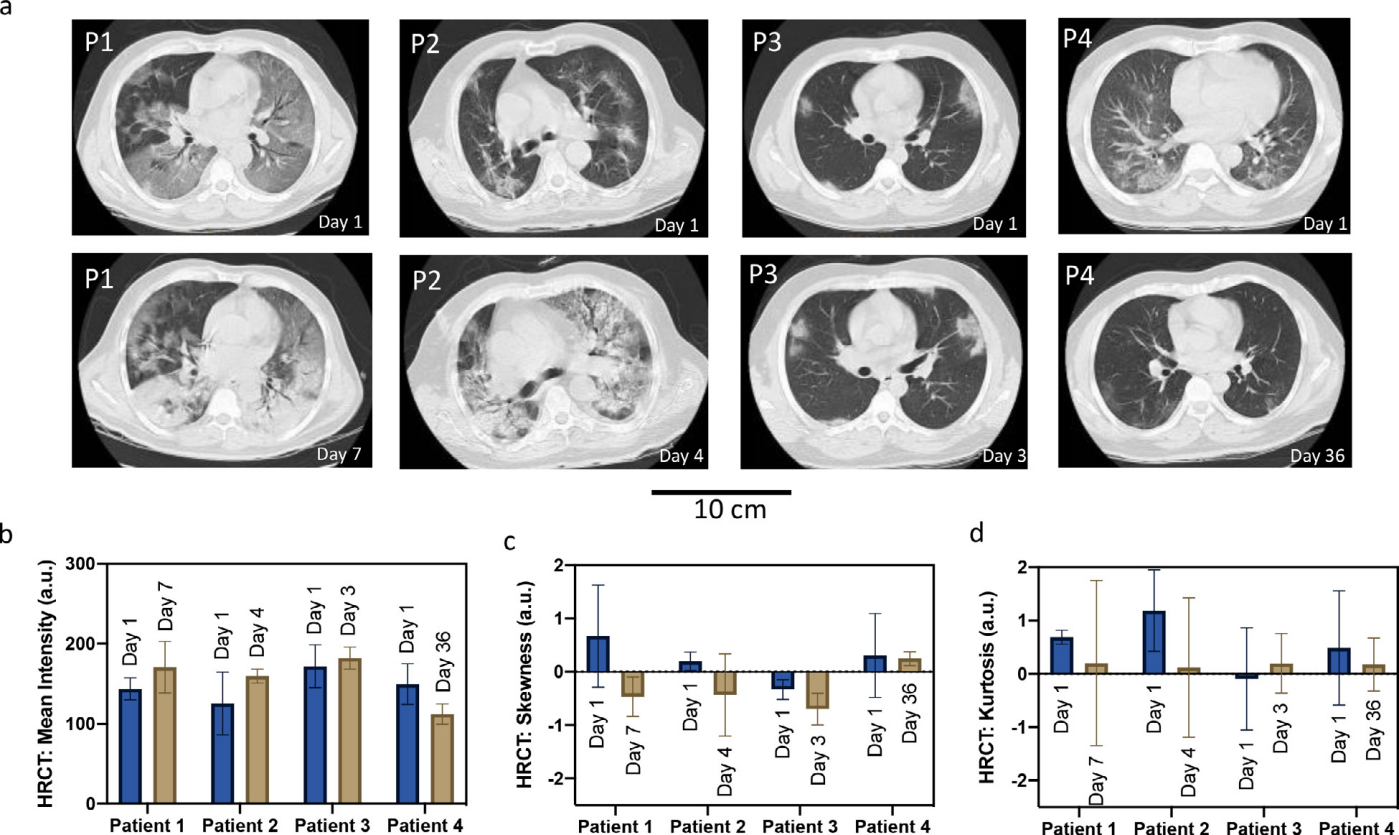
a) Transverse CT scans [P1]: patient with low dose lung HRCT showing bilateral continuous ground glass opacities with septal thickening (crazy paving appearances) more severe in left lung is an indicator COVID-19 pneumonia. After 7 days opacities became denser (alveolar consolidation). [P2]: Patient with low dose lung HRCT showing bilateral patchy ground glass opacities (GGO), suggestive of Covid-19 pneumonia. After 4 days opacities become continuous and with septal thickening (crazy paving appearances). [P3]: Patient with low dose lung HRCT showing multiple peripheral patchy GGO, typical for Covid-19 pneumonia. After 3 days, number of patches are increased and the size of patches become larger. [P4]: Patient low dose HRCT showed bilateral peripheral and posterior ground glass patches with septal thickening (crazy paving appearance), after 36 days opacities become smaller with ground glass appearance. b) intensity, c) skewness and d) kurtosis of HRCT images of four patients at different days.

Finally, in Fig. 6, the examples of COVID-19 pneumonia in different times (in different patients) during the course of infection were analyzed using HRCT images. It can be seen that there is a direct correlation between the results of image analysis and findings by the radiologists. For the patients 1 to 3 where the second images are obtained after 7, 4 and 3 days, respectively, the severity of lung damage is increased as explained with details in the figure while for the patient with second image after 36 days, the severity was significantly decreased, and the patient was recovered from hospital (see P1-4 in Fig. 6). These results are in agreement with the findings of [34], [35], which explain in the first and second weeks after symptom onset, the extent of disease on HRCT has a marked increase while it is decreased gradually in the third week.

It is worthy to mention that this analysis has been performed from the patients at the early days of COVID-19 pandemic when sufficient information and facilities were not well available for the COVID disease diagnosis. For example, at that time the RT-PCR kits were not sufficient, and it took time to provide the results (3–5 days). Additionally, due to a large number of patients visiting the hospital were suspected with COVID-19, the main goal was to provide a quick detection and avoid the spread of virus in the province. Even though the HRTC has a low specificity and it is not considered as the standard method to detect COVID-19, using HRCT as a complementary method for detection and diagnosis of COVID-19 beside RT-PCR, can compensate the low sensitivity of RT-PCR, especially where additional RT-PCR tests are required for critical patients. In long term, this approach could be improved by using advanced image analysis tools such as machine learning and artificial intelligence to enhance the low specificity of HRCT. Also, development of advanced biosensing tools, such as those which detect viruses directly or indirectly *via* antibodies, may address the specificity and sensitivity issues of HRCT and RT-PCR. Therefore, these results serve as important findings for utilizing simple analytical methods applied to clinical images for identification of risk factors associated with the disease under detection.

## 4 Conclusions

So far in the literature and clinical practice, the HRCT technique for the diagnosis COVID-19 is considered inferior to RT-PCR for the initial detection of the disease i.e. when typical symptoms of the disease start to appear. However, RT-PCR, though rapid, well-established and more sensitive than HRCT, is prone to false hits even in the severe COVID-19 cases as shown by work. We also demonstrate that for such cases, the chest HRCT has higher sensitivity than RT-PCR test (with 55.6% accuracy in detecting virus) for the diagnosis of COVID-19 pneumonia. Therefore, the most significant conclusion of our study from clinical perspective suggests that HRCT should be used as a mandatory complementary technique alongside RT-PCR for patients with severe symptoms of COVID-19 disease, especially when RT-PCR shows negative results for the disease. Moreover, the image analysis method used in our work is simple and can easily be implemented within clinical analysis protocols for HRCT to determine risk associated with the disease. Future studies could include developing open source online image analysis tool for our current methodology. The work also severs as a benchmark report for researchers developing HRCT image contrast dyes for the detection of pathogen in lungs. Several researchers developing advanced biosensors based on spike protein detection or antibody against protein detection [18] may also find our work useful for underdstanding limitations of both HRCT and RT-PCR techniques used for the detection of COVID-19. This will eventually lead to either more optimized use of the established tools in clinical practice or fast development of new sensitive tools for the diagnosis of viral diseases. The work also encourage more collaborative work between clinicians and technology development (such as image analysis tool demonstrated here) to enhance further understanding and preparedness of the COVID-19 to combat its near future variants of concern.

## CRediT authorship contribution statement

**Fariba Asadi:** Conceptualization, Methodology, Validation, Formal analysis, Writing - review & editing. **Razieh Shahnazari:** Validation, Formal analysis. **Nikhil Bhalla:** Methodology, Formal analysis, Writing - original draft. **Amir Farokh Payam:** Formal analysis, Writing - original draft.

## Declaration of Competing Interest

The authors declare that they have no known competing financial interests or personal relationships that could have appeared to influence the work reported in this paper.
